# A Decrease in Transcription Capacity Limits Growth Rate upon Translation Inhibition

**DOI:** 10.1128/mSystems.00575-20

**Published:** 2020-09-08

**Authors:** Qing Zhang, Elisa Brambilla, Rui Li, Hualin Shi, Marco Cosentino Lagomarsino, Bianca Sclavi

**Affiliations:** a LBPA, UMR 8113 CNRS, ENS Paris-Saclay, Cachan, France; b CAS Key Laboratory of Theoretical Physics, Institute of Theoretical Physics, Chinese Academy of Sciences, Beijing, China; c School of Physical Sciences, University of Chinese Academy of Sciences, Beijing, China; d LCQB, UMR 7238 CNRS, Sorbonne Université, Paris, France; University of Illinois at Chicago

**Keywords:** antibiotic, transcription, translation

## Abstract

In bacterial cells, inhibition of ribosomes by sublethal concentrations of antibiotics leads to a decrease in the growth rate despite an increase in ribosome content. The limitation of ribosomal activity results in an increase in the level of expression from ribosomal promoters; this can deplete the pool of RNA polymerase (RNAP) that is available for the expression of nonribosomal genes. However, the magnitude of this effect remains to be quantified. Here, we use the change in the activity of constitutive promoters with different affinities for RNAP to quantify the change in the concentration of free RNAP. The data are consistent with a significant decrease in the amount of RNAP available for transcription of both ribosomal and nonribosomal genes. Results obtained with different reporter genes reveal an mRNA length dependence on the amount of full-length translated protein, consistent with the decrease in ribosome processivity affecting more strongly the translation of longer genes. The genes coding for the β and β' subunits of RNAP are among the longest genes in the Escherichia coli genome, while the genes coding for ribosomal proteins are among the shortest genes. This can explain the observed decrease in transcription capacity that favors the expression of genes whose promoters have a high affinity for RNAP, such as ribosomal promoters.

**IMPORTANCE** Exposure of bacteria to sublethal concentrations of antibiotics can lead to bacterial adaptation and survival at higher doses of inhibitors, which in turn can lead to the emergence of antibiotic resistance. The presence of sublethal concentrations of antibiotics targeting translation results in an increase in the amount of ribosomes per cell but nonetheless a decrease in the cells’ growth rate. In this work, we have found that inhibition of ribosome activity can result in a decrease in the amount of free RNA polymerase available for transcription, thus limiting the protein expression rate via a different pathway than what was expected. This result can be explained by our observation that long genes, such as those coding for RNA polymerase subunits, have a higher probability of premature translation termination in the presence of ribosome inhibitors, while expression of short ribosomal genes is affected less, consistent with their increased concentration.

## Abstract (precis)

Exposure of bacteria to sublethal concentrations of antibiotics can lead to bacterial adaptation and survival at higher doses of inhibitors, which in turn can lead to the emergence of antibiotic resistance. The presence of sublethal concentrations of antibiotics targeting translation results in an increase in the amount of ribosomes per cell but nonetheless a decrease in the cells’ growth rate. In this work, we have found that inhibition of ribosome activity can result in a decrease in the amount of free RNA polymerase available for transcription, thus limiting the protein expression rate via a different pathway than what was expected. This result can be explained by our observation that long genes, such as those coding for RNA polymerase subunits, have a higher probability of premature translation termination in the presence of ribosome inhibitors, while expression of short ribosomal genes is affected less, consistent with their increased concentration.

## INTRODUCTION

Bacteria often encounter sublethal levels of antibiotics produced by other microorganisms in their environment. A decrease in growth rate under these conditions can allow a strain to survive long enough until the inhibitor is no longer present or, in some cases, until the bacteria become resistant to the antibiotic via the selection of preexisting mutations or an increase in mutation rates (1–4). However, the mechanistic details of these response pathways often remain to be described. The cellular response to the limitation of translation activity is thought to be related to the pathway involved in the stringent response, the regulatory mechanism that decreases ribosome production in response to a decrease in amino acid availability. This is mediated by the change in concentration of the secondary messenger molecule, (p)ppGpp, that is produced by the RelA enzyme when the pool of amino acids decreases and ribosomes are not loaded with charged tRNAs (5, 6). ppGpp can directly inhibit ribosome assembly (7, 8) and the activity of RNA polymerase (RNAP) at ribosomal promoters while increasing the activity of the promoters of genes for amino acid biosynthesis (9). The transcription of ribosomal operons can use a large fraction of the free RNA polymerase pool in the cell because of the high affinity of the ribosomal promoters for the enzyme and a high frequency of transcription initiation. Therefore, the regulation of ribosomal promoter activity can be a means by which the pool of free RNA polymerase can be repartitioned between ribosomal operon transcription and nonribosomal mRNA synthesis. This has been referred to as the “passive control” of transcription regulation (10–14).

The ppGpp-dependent feedback loop also plays a role in the regulation of ribosome content as a function of growth rate (15–17). Growth rate-dependent regulation of gene expression determines the allocation of cellular resources between the production of ribosomes and that of other proteins and results in a linear increase in ribosome content with increasing growth rate (15, 18–20). In richer growth media, when the amount of amino acids is higher, ppGpp levels are lower, favoring ribosome production and a higher fraction of active ribosomes (16). In poorer media, it is the inverse: accumulation of ppGpp slows down the production of new ribosomes, and a smaller fraction of the ribosome pool is in an active form (19).

When ribosome activity is inhibited by sublethal concentrations of antibiotics, amino acids are used more slowly and their concentration increases, which can result in a decrease in the intracellular ppGpp pool (5). The cellular response, as predicted by the ppGpp feedback loop, is to produce a larger amount of ribosomes and an increased translation rate; however, despite this increase, the cell's growth rate is reduced (18, 19). This has been proposed to result from a decrease in the resources available for the production of nonribosomal proteins that become limiting for cellular metabolism (18). More recent results point to a decrease in the fraction of active ribosomes to explain the decrease in the total protein production rate (19, 21).

To measure the effect that the inhibition of ribosome activity can have on gene expression resulting from a possible repartition of RNAP, we have compared the activity of a ribosomal promoter to that of constitutive promoters with different affinities for RNAP. This approach stems from a well-established protocol developed by Hans Bremer and coworkers of using quantitative measurements of changes in constitutive and ribosomal promoter activity as reporters of changes in the amount of free RNAP and of ppGpp (22–25).

In parallel, we analyzed transcriptomics and proteomics data from the literature on the direct and indirect effects of changing ppGpp concentration and translation limitation on gene expression (26, 27). The results from this analysis are consistent with a linear decrease with decreasing growth rate in the concentration of free RNAP available for promoter binding and transcription. We propose a model that can explain this decrease in transcriptional activity based on the observation that gene length and a gene’s operon position can be an important parameter on the change in protein expression in the presence of sublethal levels of chloramphenicol and that two of the four proteins composing the core of RNAP are two of the longest gene products in the Escherichia coli genome that, in addition, are found within the same operon.

## RESULTS

### Transcription regulation by ppGpp does not suffice to explain gene expression changes with increasing translation inhibition by chloramphenicol.

**FIG 1:**
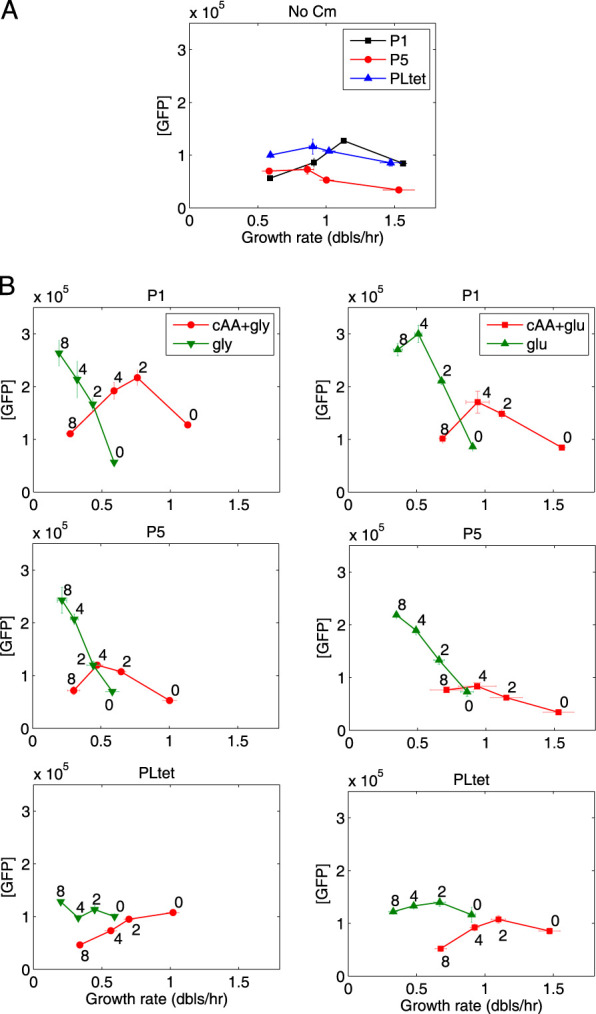
Promoters with different affinities for RNAP react differently to translation limitation. (A) Change in GFP concentration (relative fluorescence units [RFU] of GFP/OD_610_) as a function of growth rate (in doublings per hour). Four growth media were used, from the slowest to the fastest: M9-glycerol, M9-glucose, M9-glycerol+Casamino Acids, M9-glucose+Casamino Acids. P5 and PLtet are both constitutive promoters with different affinities for RNAP, while P1 is a shortened version of the *rrnB*P1 rRNA promoter with a RNAP affinity similar to P5 but regulated by ppGpp. (B) Change in GFP concentration as a function of increasing concentration of chloramphenicol in the four growth media. The growth media with Casamino Acids (cAA) are shown in red, and the ones without Casamino Acids are in green. The four points correspond to 0, 2, 4, and 8 μM final chloramphenicol concentration as noted next to the data points. The error bars represent the standard errors of the means (SEM) from 3 independent experiments. The error bars smaller than the symbols are not shown. Comparison of the panels shows that ppGpp regulation at the transcriptional level alone cannot account for the change in GFP expression in response to translation limitation.

In order to measure the effect of increasing ribosome inhibition on RNAP repartition between ribosomal and nonribosomal promoters, we have chosen three reporter cassettes. The first contains a shortened version of the well-characterized rRNA operon promoter *rrnB*P1, here called P1, that includes the sequence from −69 to +6 relative to the transcription start site (28). The binding sites for Fis and the higher-affinity H-NS binding site are thus omitted from this construct. This promoter has a GC-rich discriminator region at the transcription initiation site that makes the open complex sensitive to changes in negative supercoiling and to inhibition by ppGpp (see Table S1 in the supplemental material) (24, 29, 30). The second promoter used here is a constitutive promoter, P5, that has consensus −10 and −35 sequences and no discriminator region. The third is PLtet, also a strong constitutive promoter with no discriminator region but with a lower affinity for RNA polymerase due to a nonconsensus −10 sequence (31) (Table S1). Bremer and coworkers have shown that the activity of the *rrnB*P1 promoter is inversely proportional to the concentration of ppGpp *in vivo* (24) and that the activity of constitutive promoters can be used to estimate the amount of free RNA polymerase in the cell (22, 24). Each of these promoters was placed upstream of the *gfpmut2* gene, and this cassette was inserted in the chromosome together with a kanamycin resistance gene expressed divergently from the chosen promoter (see Fig. S1 in the supplemental material). Growth of these strains in a 96-well plate allowed us to measure the changes in growth rate, the green fluorescent protein (GFP) concentration, and the resulting GFP production rate (Gpr) as a function of chloramphenicol concentration (Fig. 1 and Fig. S1). We compared four different growth media, M9 with glucose (M9-glu), M9 with glycerol (M9-gly), and these two media supplemented with Casamino Acids (cAA). This results in four different growth rates. Furthermore, it has already been shown that cells growing in a growth medium containing amino acids have a lower concentration of ppGpp (16, 24), allowing us to compare the effects due to changing concentrations of this key metabolite without the use of mutant strains that can result in secondary effects on cell metabolism due to the multiple targets of ppGpp (8, 32).

10.1128/mSystems.00575-20.2 FIG S1 Schematic diagram of the experimental designs and data analysis to measure bacterial growth rate and the concentration of the reporter protein. (A) Chromosomal insertion of the reporter constructs. The reporter gene *gfpmut2* or *lacZ* is fused downstream of a specific promoter (PLtet, P5, or P1) and then inserted in *E.coli* chromosome near the gene *crl*, *cynR*, or *uspE*. A kanamycin resistance gene expressed divergently was inserted into the chromosome together with the constructs. (B) The strains carrying GFP were cultivated in a 96-well plate, and their optical density (OD) at 610 nm and green fluorescence were measured with a plate reader (Tecan Infinite 200Pro) every 7 min for at least 30 h. The growth rate μ was derived from the slope of the plot of log OD versus time over the exponential phase, which is defined between a lower and an upper threshold value of OD. GFP concentration ([GFP]) was derived from the slope of the plot of GFP versus OD over the exponential phase. For comparison, bacteria with P5-*gfpmut2* and bacteria with P5-*lacZ* were grown in flasks, and OD and fluorescence were measured with the plate reader every 30 to 50 min. See Li et al. (39) for the RFP-GFP operon system shown in Fig. 4. (C) The strains carrying β-galactosidase (β-gal) were grown in flasks, and the ODs were measured in 96-well plates with the plate reader. The growth rate was obtained in the same way as for the GFP strains. β-gal concentration ([β-gal]) was derived from the 96-well β-gal assay (see details in Zhang et al. [60]). The red star indicates the time when the culture sample was taken for the β-galactosidase assay. Download FIG S1, EPS file, 0.6 MB. Copyright © 2020 Zhang et al. 2020 Zhang et al. This content is distributed under the terms of the Creative Commons Attribution 4.0 International license.

10.1128/mSystems.00575-20.10 TABLE S1 Sequences of the promoters used in this study. The −35 and −10 regions are underlined. The bold base is the transcription start site. The bases in red in the P1 sequence indicate the discriminator region. Download Table S1, XLSX file, 0.01 MB. Copyright © 2020 Zhang et al. 2020 Zhang et al. This content is distributed under the terms of the Creative Commons Attribution 4.0 International license.

In the absence of translation inhibition, the change in promoter activity measured as a function of growth rate is consistent with previous measurements on constitutive promoters and *rrnB*P1-derived promoters (11, 28, 33) (Fig. 1A). The concentration of GFP from the constitutive promoters tends to decrease at the higher growth rates, due to their lack of specific growth rate-dependent regulation and the increased dilution rate (34), while the concentration of GFP expressed from the *rrnB*P1 promoter increases with growth rate until the last point, where Fis activation, absent in this construct, has been shown to be required for continued increased expression (28). The PLtet promoter has a lower affinity for RNAP than P5 does (see below); however, when RNAP binds at the PLtet promoter, it initiates transcription with a higher frequency than at P5 (31), resulting in a higher promoter activity and consequently a higher GFP concentration (Fig. 1A).

Previous work has shown that as the concentration of chloramphenicol is increased, the total RNA content relative to the total protein mass increases—reflecting the increase in rRNA—and the concentration of a reporter protein expressed from a constitutive promoter decreases (18). Therefore, the expected result here is that the GFP concentration from a rRNA promoter (P1) should increase while the concentration from a constitutive promoter (P5 or PLtet) should decrease, with the lower-affinity constitutive promoter (PLtet) decreasing at a higher rate if increasing amounts of RNAP are being used for transcription of ribosomal operons. The comparison of P1 and PLtet agrees with this prediction. Unexpectedly, however, for the P1 and P5 promoters, the patterns of change in GFP production rate and GFP concentration are very similar (Fig. 1 and Fig. S2).

10.1128/mSystems.00575-20.3FIG S2Estimated change in free RNAP concentration from the ratio of GFP production rates from the PLtet and P5 promoters, and estimated change in ppGpp effect on P1 transcription and ppGpp concentration from the ratio of GFP production rates from the P1 and P5 promoters. (A) Measured ratio of GFP production rates from PLtet and P5 as a function of growth rate. The symbols indicate the experimental data. The black line denotes the equation of the ratio of transcription rates from PLtet and P5 without Cm, i.e., *a*(*c_f_* + *K*_5_)/(*c_f_* + *K*_Ltet_), where *c_f_* is the free RNAP concentration and *a*, *K*_5_, and *K*_Ltet_ are constants which can be determined by fitting the ratios of GFP production rate to the known values of free RNAP concentrations as a function of growth rate. The free RNAP concentrations as a function of growth rate in the absence of Cm was estimated based on a double exponential function shown by the black line in panel B. (B) The black stars correspond to the estimated free RNAP concentration as a function of growth rate obtained from Klumpp and Hwa (34). These data are fit with a double exponential function *c_f_* = exp [*A*exp(–μ_r_/μ)] (black line), where μ denotes the growth rate, *c_f_* denotes the concentration of free RNAP, and *A* and μ_r_ are constants. The free RNAP concentration in the presence of Cm is estimated from the data in panel A with the transcription rate ratio mentioned above. (C) The relative ppGpp inhibition effect is estimated as the ratio of GFP production rates from P1 and P5 promoters divided by the ratio of their relative promote activities, i.e., (*K*_5_ + *c_f_*)(*K*_1_ + *c_f_*), where *c_f_* denotes the free RNAP concentration and *K*_5_ and *K*_1_ are the dissociation constants for P5 and P1, respectively. The black line indicates a Hill function, i.e., $bk_{p}^{n}/(k_{p}^{n}+c_{p}^{n})$, where *c_p_* is ppGpp concentration and *b*, *k_p_*, and *n* are constants which are fixed by fitting the estimated ppGpp effect without Cm. ppGpp concentration at the growth rates without Cm was estimated based on an exponential function shown by the black line in panel D. (D) The black stars correspond to the data on ppGpp concentration as a function of growth rate (20). These data are fit with a function *c_p_* = *c_p_*_0_ exp(–μ/μ*_p_*) (black line), where μ denotes the growth rate, *c_p_* denotes the concentration of ppGpp, and *c_p_*_0_ and μ*_p_* are constants. ppGpp concentration in the presence of Cm is estimated from the data in panel C with the Hill function mentioned above. See details of the analysis in Materials and Methods. Download FIG S2, EPS file, 0.5 MB.Copyright © 2020 Zhang et al.2020Zhang et al.This content is distributed under the terms of the Creative Commons Attribution 4.0 International license.

The pattern of the change of expression for these two promoter constructs depends strongly on whether the growth medium contains Casamino Acids, independently of the carbon source. In the absence of cAA, the increase in GFP concentration as a function of chloramphenicol (Cm) is significantly greater than in their presence (Fig. 1). Increased expression from the P1 promoter in the growth media lacking cAA would be expected from a decrease in ppGpp as a result of increased amino acid pools due to ribosome inhibition; the P5 promoter on the other hand does not contain the GC-rich discriminator region and is not expected to show increased activity upon a decrease in ppGpp. ppGpp, however, is also known to negatively regulate the activity of ribosomes and the overall translation rate as well as the transcription elongation rate (7, 8, 21); therefore, a decrease in ppGpp in the growth media lacking cAA is expected to lead to an increase in translation rate, irrespective of the promoter sequence. The similar increase in GFP concentration of these two different promoters thus points to a stronger effect of ppGpp on GFP expression at the level of translation and transcription elongation of the *gfpmut2* gene rather than at the transcription initiation level. In summary, regulation of transcription initiation by ppGpp cannot solely explain the change in GFP expression from different promoters with increasing sublethal levels of chloramphenicol. However, the shared increase in GFP concentration observed for both the P1 and P5 promoters is consistent with an overall increase in the translation rate, as observed previously in these experimental conditions (19, 21).

### A decrease in free RNAP concentration with increasing translation inhibition by chloramphenicol.

**FIG 2:**
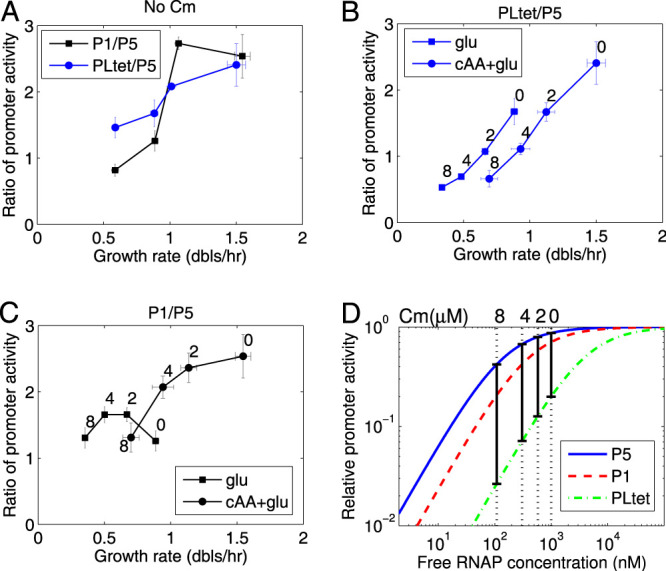
Ratios of promoter activities for the different promoters can be used to estimate the changes in the concentration of free RNAP. (A) Ratio of PLtet to P5 and P1 to P5 as a function of growth rate (in doublings per hour). (B) Ratio of PLtet to P5 with increasing chloramphenicol concentration. (C) Ratio of P1 to P5 with increasing chloramphenicol concentration. (D) Estimated decrease in the concentration of free RNAP from the change in the ratios of promoter binding as a function of chloramphenicol concentration. To obtain this estimate, the relative activity of the promoters as a function of free RNAP concentration (*c_f_*) was obtained from the respective RNAP binding constants, *K_i_* ∈ {*K*_1_, *K*_5_, *K*_Ltet_} using *c_f_*/(*K_i_* + *c_f_*). The constant *K*_5_ is determined by fitting the data obtained in the absence of Cm from our experiments and the amount of free RNAP obtained from the literature (34) (see Materials and Methods). *K*_1_ and *K*_Ltet_ were derived by the formula *K*_5_ exp(Δ*E*), where Δ*E* is the difference between the binding energies of RNAP with P1 (or PLtet) and P5 (see Materials and Methods). The black vertical bars indicate the concentrations of free RNAP that give the measured promoter activity ratios shown in panel B for different Cm concentrations, shown above the plot. The ratio of PLtet and P5 promoter activities for example is obtained by taking the ratio of GFP production rates (Gpr): Gpr(PLtet)/Gpr(P5) = *a*(*K*_5_ + *c_f_*)/(*K*_Ltet_ + *c_f_*), where *a* is a scaling factor. *K*_5_ and *a* were fixed by fitting the data in the absence of Cm from our experiments and the literature (34) (see Materials and Methods). Data from the cells growing in cAA-glu was used so that ppGpp-dependent regulation of P1 is small and can be ignored. Fig. S2A and B in the supplemental material shows the estimation of RNAP concentration for the four growth media used. Fig. S2C and D shows an estimation of the change in ppGpp as a function of chloramphenicol concentration.

Since the translation rate of GFP is shared by the three promoter constructs, it is possible to obtain an estimate of the magnitude of the promoter-specific effect of ppGpp, and of changes in free RNAP, on the transcription rate by measuring the ratios of GFP production rates. This operation “cancels out” the translation component of gene expression and isolates the transcription-specific effect as the ratio of promoter activities (see Materials and Methods). Figure 2A shows the change in the ratios of promoter activities as a function of growth rate in the absence of chloramphenicol. The ratio of P1 to P5 rates increases rapidly between M9-glu and M9-cAA-gly, consistent with a lower level of ppGpp in the cells growing in the presence of cAA (24) increasing the probability of transcription initiation specifically from P1.

In the presence of Cm, the fold increase of gene expression from P1 is greater than the one of P5 in the cells that are grown without cAA, consistent with a decrease in ppGpp levels by the addition of the antibiotic (Fig. 2C). As the Cm concentration is increased further, the difference between the two promoters decreases again to the initial level. On the other hand, in the growth media with cAA, and thus lower levels of ppGpp, the P1-to-P5 ratio decreases, indicating that the change in GFP production rate from P1 is lower than that of P5 as a function of increasing Cm. A similar result is also observed for the PLtet-to-P5 ratio, this time independently of either the carbon source or amino acid content (Fig. 2B).

The comparison of two constitutive promoters with differing affinities for RNAP can be used to estimate the change in the amount of free RNAP that is available for transcription *in vivo* (24, 35). Since PLtet has a lower affinity for RNAP than P5, if the free RNAP concentration increases, the ratio of promoter activities of PLtet and P5 will increase when the amount of free RNAP is within a range of concentrations that span the dissociation constant of PLtet (Fig. 2D). In this same range of concentrations, the binding of RNAP to P5 will change by a smaller amount, since the higher affinity of this promoter means that it will be almost at saturation. This is indeed what is observed in the data as a function of growth rate in the absence of Cm (Fig. 2A), in agreement with the previous estimates of the change in free RNAP as a function of doubling time (23, 34).

The change in activity of the promoters as a function of free RNAP concentration (*c_f_*) can be obtained from the respective RNAP binding constants, *K_i_*∈{*K*_1_, *K*_5_, *K*_Ltet_} using *c_f_*/(*K_i_* + *c_f_*). The differences in binding affinity for each promoter can be estimated based on a statistical-mechanical selection model developed by Berg and von Hippel (36) where Δ*E* is the difference between the binding energies (see Text S1 in the supplemental material). If one of these affinity constants is known, one can then use these calculations to estimate the other two. The affinity of RNAP for P5 in terms of RNAP concentration can be obtained from fitting the change in the Gpr(PLtet)/Gpr(P5) ratio as a function of growth rate from our experimental data (Fig. 2A and Fig. S3A) with the following equation, Gpr(PLtet)/Gpr(P5) = *a*(*K*_5_ + *c_f_*)/(*K*_Ltet_ + *c_f_*), where *a* is a scaling factor that accounts for the difference in transcription initiation frequency, and the RNAP concentration at the different growth rates is obtained from a previous study (34) (see Materials and Methods).

10.1128/mSystems.00575-20.1 TEXT S1 Supplemental information including text describing the following: (i) estimation of the affinity of RNAP-promoter interaction from the promoter sequence, (ii) estimation of the change in ppGpp concentration from the analysis of the effect of Cm on GFP expression from the P5 and P1 promoters, (iii) estimation of the change in transcription and translation rates as a function of Cm, (iv) a quantitative model of the probability of a stalled ribosome as a function of gene length and as a function of chloramphenicol concentration, (v) analysis of previously published data sets showing that a decrease in translation processivity can result in decreased expression of late operon genes, (vi) quantitative model of the probability of a stalled ribosome leading to mRNA degradation that can explain the effect of chloramphenicol on gene expression as a function of gene position in an operon, and (vii) the role of the discriminator region in determining changes of gene expression under translation limiting conditions. Download Text S1, PDF file, 0.09 MB. Copyright © 2020 Zhang et al. 2020 Zhang et al. This content is distributed under the terms of the Creative Commons Attribution 4.0 International license.

**FIG 3:**
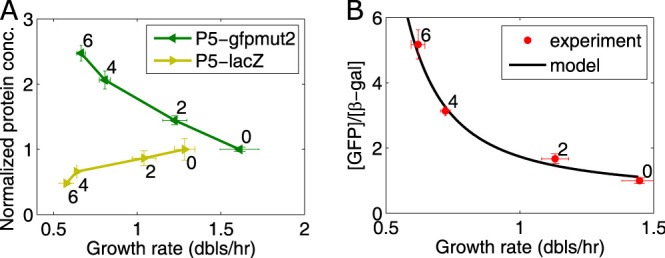
Gene length can influence gene expression under translation limitation. (A) Change in GFP (238 aa) and β-galactosidase (1,024 aa) expressed from the P5 promoter as a function of increasing chloramphenicol concentration. The bacteria were grown in M9 with glucose plus cAA with chloramphenicol (Cm) at concentrations of 0, 2 μM, 4 μM, or 6 μM in flasks. The concentrations were normalized by dividing the data by the point without Cm. (B) Fit of the model to the ratio of GFP to β-galactosidase concentrations from the data in panel A. The growth rate at each chloramphenicol concentration is the average over the two strains. The error bars correspond to the SEM from three independent experiments.

10.1128/mSystems.00575-20.4 FIG S3 Fitting experimental data with the model. (A) Determination of β0 by fitting experimental data of Tsung et al. (37) in the absence of Cm with the model. The proteins of different lengths shown by the circles were expressed from the multimers of the *lacZ* gene (only the last one confers β-galactosidase activity). The activities of all the proteins were divided by the activity of the wild-type protein. (B) The dependence of growth rate on the Cm concentration can be fit with equation 18 (in Text S1) that we derived based on the work of Dai et al. (19). (C) The dependence of growth rate on the Cm concentration can be fit with the formula derived by Greulich et al. (44). (D) The concentration ratio between GFP and β-galactosidase as a function of growth rate can also be fit with the model when the growth rate-Cm formula of Greulich et al. (44) is used. The experimental data used in panels B to D are the same as those shown in Fig. 3B. Download FIG S3, EPS file, 0.2 MB. Copyright © 2020 Zhang et al. 2020 Zhang et al. This content is distributed under the terms of the Creative Commons Attribution 4.0 International license.

Figure 2D shows the estimated binding curves for RNAP to each promoter; the vertical bars indicate the ratio of promoter activity that corresponds to the values measured experimentally (Fig. 2B). The Cm concentration for each of these ratios is shown on the top *x* axis. The positions of these black vertical bars relative to the *x* axis indicate the concentrations of free RNAP.

Because of their different affinities for RNAP, the decrease in free RNAP with increasing chloramphenicol has a stronger effect on PLtet first, then on P1, and finally on P5. Note that in order for the ratios obtained from the experimental results to be coherent with the RNAP binding curves, the P5 promoter needs to be nearly saturated by RNAP in the absence of Cm, consistent with the high affinity of the interaction resulting from the consensus −10 and −35 sequences of this promoter. This simple model of RNAP-dependent capacity can explain the data in Fig. 2B on the change in the PLtet-to-P5 ratio.

Therefore, from the values of the RNAP affinities for the two constitutive promoters, PLtet and P5, and the relative changes in promoter activity, it is possible to estimate the change in the amount of free RNAP in the cells as a function of increasing translation limitation. Figure S2 in the supplemental material shows the estimation of RNAP concentration obtained for the four growth media used here.

Using the ratio of the promoter activities of P1 and P5, it is possible to estimate the change in ppGpp as a function of growth rate and as a function of Cm (Fig. S2), in a fashion similar to the approach validated by Bremer and colleagues (24).

In summary, the changes in transcription activity measured by the decrease in the PLtet-to-P5 and P1-to-P5 ratios are in line with a decrease in free RNAP concentration. This decrease is independent of a ppGpp-mediated repartition between ribosomal and nonribosomal promoters, since it can be observed to the same extent in the growth media with and without cAA (Fig. 2B and Fig. S3), which have been previously shown to result in different ppGpp levels in the cell (16).

### The decrease in ribosome processivity by chloramphenicol reduces the expression of longer genes more than shorter ones.

A possible cause for the decrease in the pool of free RNAP independently of the changes in ppGpp is a decrease in the total amount of RNAP per cell. The RNA polymerase holoenzyme is composed of five subunits: β, β′, ω, two copies of α, and a σ factor. *rpoB* and *rpoC*, coding for β and β′, are among the longest genes in E. coli with a length of 4,029 bp and 4,224 bp, respectively (the average gene length in E. coli is about 900 bp [see Fig. S6]).

**FIG 4:**
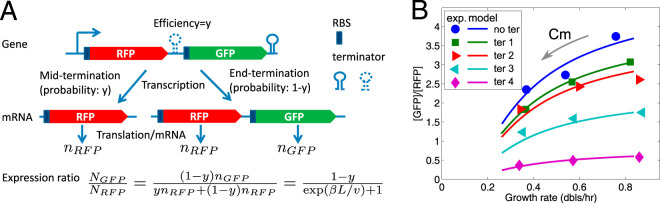
Operon position can influence gene expression under translation limitation; however, it is independent of transcription-translation coupling. (A) Sketch for GFP and RFP fused in an operon with an intergenic terminator of efficiency *y* (see the details of the construction in reference 39) and the formula of the expression ratio of GFP to RFP. When the efficiency *y* is zero, it corresponds to the case without intergenic terminator (see “no ter” in panel B). (B) The expression ratio between downstream GFP and upstream RFP [in the units of RFU(GFP)/RFU(RFP)], as a function of growth rate decreases with increasing Cm concentration. The experimental ratios (symbols) are fit with the same model (lines) on ribosome stalling-induced mRNA degradation used to describe the difference between β-gal and GFP expression in Fig. 3 (see supplemental material). “no ter” and “ter 1 to 4” correspond to terminator sequences of increasing efficiency that can be used to estimate the contribution of transcription-translation coupling (“I21” [no terminator], “R9,” “R17,” “W13,” and “R32,” respectively, in reference 39). The cells were grown in M9 minimal medium containing glucose. Cm was added to a final concentration of 0, 2, or 4 μM.

10.1128/mSystems.00575-20.5 FIG S4 Increasing concentration of chloramphenicol result in the same change in GFP/RFP independently of the efficiency of the terminator. Fractional change in GFP/RFP as a function of growth rate with increasing Cm concentrations (0, 2, and 4 μM), from the data in Fig. 4 (normalized by the point without Cm for each construct). Download FIG S4, EPS file, 0.1 MB. Copyright © 2020 Zhang et al. 2020 Zhang et al. This content is distributed under the terms of the Creative Commons Attribution 4.0 International license.

10.1128/mSystems.00575-20.6 FIG S5 A subset of R-sector protein expression is regulated by ppGpp. (A) Schematic diagram of the divergent change of R-sector and P-sector protein concentrations under translation limitation based on reference 18. The difference in the fold change of a given protein as a function of inhibitor can be described by its slope. A negative slope means that the concentration of one protein increases with increasing inhibitor concentration, whereas a positive slope means it decreases. The slope for R-sector protein is –tan(α) < 0, and for P-sector protein, it is tan(β) > 0. Q-sector proteins are those whose concentration is not changed by the translation inhibitor. The pies were adapted from Scott et al. (18). (B) Venn diagram showing the intersection between the genes whose expression is inhibited (directly or indirectly) by changes in (p)ppGpp concentration (27), R-sector proteins, and other sector proteins identified by mass spectrometry (26). The hypergeometric *P* value for overrepresentation of the intersection between the blue and red sets is 1.5 × 10^−32^, and the *P* value for underrepresentation of the intersection between the red and green sets is 0.012. (C) Distribution of the similarity of the −8 to −1 promoter sequence compared to the consensus sequence of the discriminator region. In producing the distribution of random sequences, the bias in base usage of E. coli genome (GC content = 43.4%) was considered. The Kolmogorov-Smirnova test for the data set of the R sector inhibited by ppGpp and that of R sector uninhibited by ppGpp plus other sectors gave a two-sided *P* value of 0.005. Download FIG S5, EPS file, 0.3 MB. Copyright © 2020 Zhang et al. 2020 Zhang et al. This content is distributed under the terms of the Creative Commons Attribution 4.0 International license.

10.1128/mSystems.00575-20.7 FIG S6 Genes whose product belongs to the R sector tend to have promoters with a higher affinity for RNAP, and most of the short R-sector proteins are ribosomal proteins. The list of ribosomal proteins are obtained from Ecocyc (64). (A and B) The distribution of RNAP-promoter binding energy as a function of protein sector and gene regulation. The method of Berg and von Hippel (36) considering the spacer length effect was used to estimate promoter affinity for RNAP (see details in Materials and Methods). The plots compare the RNAP affinities of the promoters in the R sector (R) and other sectors (NR) over those whose affinity is calculable. From these two sets, R and NR, two new subsets are defined, whether the promoters are constitutive (as defined by Shimada et al. [65]), or whether they are neither regulated by transcription factor (TF) (based on the regulatory network of all TFs from RegulonDB [66]) nor inhibited by ppGpp (based on the transcriptome of Traxler et al. [27]). The number of promoters in each subset is shown in parentheses. When ribosomal proteins are included in the analysis, a gene in R sector has on average a higher affinity for RNAP than another gene in other sectors over the three data sets (A). When ribosomal proteins are excluded from the analysis, a nonribosomal gene in the R sector has on average a higher affinity for RNAP than another nonribosomal gene in other sectors over the three data sets (B). (C and E) Gene length distribution as a function of protein sector and ppGpp regulation. The sets defined by the intersection of the data of Hui et al. (26) and Traxler et al. (27) (Fig. S5B and C) were used to calculate the probability density function as a function of gene length (C). R, R-sector proteins; NR, proteins that are not in the R sector. Panel E shows the same analysis without taking into consideration the set of ribosomal proteins. (D and F) The slope of the change in protein concentration as a function of increasing Cm calculated as in Fig. S5A as a function of gene lengths from the Hui et al. data set (26). The red line is the median, the red cross indicates the outliers. Panel F shows the same analysis without taking into consideration the set of ribosomal proteins. Download FIG S6, EPS file, 0.8 MB. Copyright © 2020 Zhang et al. 2020 Zhang et al. This content is distributed under the terms of the Creative Commons Attribution 4.0 International license.

The processivity of translation, the probability of the ribosome reaching the end of the gene before stalling and falling off, has been shown to decrease exponentially with increasing gene length (37). We reasoned that if, in addition, ribosome processivity is decreased by an inhibitor, then the probability to finish the translation of a long gene would be lowered even more compared to a shorter gene, decreasing the rate of expression of the longer gene to a greater extent in the presence of translation inhibitors.

From the known values for the affinity of ribosomes for chloramphenicol, we derived a model to calculate the probability *P*_hit_ that a ribosome will be “hit” by chloramphenicol before reaching the end of a mRNA of a given length (as described by Dai et al. [19] [see details in Text S1]). Ribosome stalling leads to the nonsymmetric degradation of mRNA and stops further translation by other ribosomes. The following equation can be used to describe the dependence of *P*_hit_ on protein length, i.e.,

(1) $$P_{\text{hit}}=1-\text{exp}\left(-k_{\text{on}}[\text{Cm}]L/v\right)$$

where *k*_on_ denotes the binding constant of Cm with ribosome [*k*_on_ = 0.034 (μM·min)^−1^ (38)], *L* indicates protein length, and *v* is the translation elongation rate dependent on the RNA/protein mass ratio (Text S1).

To test this model, we compared the changes in gene expression from the same constitutive promoter, P5, of two different genes, *gfpmut2* (714 bp), and *lacZ* (3,072 bp), in the presence of increasing chloramphenicol concentrations (Fig. 3A). Using equation 1, it is possible to estimate an order of magnitude of the effect of the antibiotic. At 8 μM Cm (the highest concentration used here), *P*_hit_ is 23% for LacZ (1,024 amino acids [aa]), while for GFP, it is 6% (238 aa).

The experimental results show that while the concentration of GFP increases as a function of Cm concentration, β-galactosidase concentration decreases (Fig. 3A). Figure 3B shows the change in the ratio of the shorter protein to the longer protein as a function of Cm concentration. The black line shows the fit obtained to the model of ribosome processivity. These results therefore indicate that a gene’s length, in addition to its promoter’'s affinity for RNAP, can influence how its expression levels change in the presence of sublethal concentrations of ribosome inhibitors and could thus explain a possible mechanism that leads to decreased RNAP availability.

By the same mechanism, increased probability of ribosomes stalling in the presence of chloramphenicol can also lead to degradation of a whole operon’s RNA, so that genes that are found farther downstream from the transcription start site have a higher probability of not being translated than those at the beginning of the operon. A recent study has shown this to be the case for the genes within the *lac* operon and those in the ribosomal protein operons S10 and Spc (21). The *rpoB* and *rpoC* genes follow each other within the same operon, which can result in a further decrease in the expression of the downstream gene.

### A decrease in translation processivity can result in decreased expression of late operon genes.

In order to obtain a quantitative measure of the effect of operon position on gene expression in the presence of translation limiting antibiotic concentrations, we have measured the expression of two fluorescent reporter proteins, red fluorescent protein (RFP) and GFP, whose genes have been placed within the same operon, where the *rfp* gene is upstream of the *gfp* gene (Fig. 4). Increasing Cm results in a decrease in the GFP-to-RFP ratio, consistent with increased polarity effects leading to premature transcription termination (21) and to degradation of the operon mRNA decreasing the expression of GFP when a ribosome translating the upstream RFP gene is inhibited by chloramphenicol. These results can be reproduced by a model using the parameter values for the probability of translation termination obtained from the comparison in GFP versus LacZ translation (Fig. 3) (see Text S1 for the details of the model).

The insertion of terminators of different efficiencies between the two genes can be used to test for the role of an interaction between RNAP and the leading ribosome in affecting the probability of transcription termination (39) (Text S1). The presence of a ribosome trailing the RNAP inhibits formation of the hairpin and therefore of transcription termination. The results obtained here show that the decrease in the GFP-to-RFP ratio by chloramphenicol is independent of the presence and efficiency of the terminator of the two genes (Fig. 4B and Fig. S8). Therefore, at these low Cm concentrations, the probability of decoupling of transcription and translation, of hitting the first translating ribosomes, is not significant enough to result in increased transcription arrest by the folding of the hairpin loop of the terminator.

Finally, in light of these results we have also analyzed data from a previously published proteomics study that measured protein fractional abundance as a function of increasing sublethal chloramphenicol concentration for a set of more than 1,000 E. coli proteins (26). We have found that by itself RNAP affinity for a given promoter is not a predictor of whether the expression of a gene will increase or decrease under translation limitation. This is because RNAP affinity for a promoter is not only dictated by the promoter’s sequence but also by the presence of possible transcription factors. It is interesting to note however that ribosomal promoters all have a higher than average affinity for RNAP, independently of the activity of their transcription activator Fis whose activity is dependent on growth rate and growth phase (Fig. S6).

The data set from Hui et al. (26) was also used to determine whether gene length could be a predictor of changes in gene expression under translation limitation (Fig. S6). Once the short ribosomal genes have been omitted from the set, gene length by itself is not enough to predict changes of gene expression, likely due to compensatory effects from differing promoter affinities for RNAP. However, when the expression of genes within the same operon, and thus likely to share the same promoter, is compared, we see that on average a downstream gene within an operon decreases more in expression than the genes found upstream within the same operon in the presence of increasing levels of chloramphenicol (Text S1 and Fig. S7).

10.1128/mSystems.00575-20.8 FIG S7 A gene’s operon position can have an effect on the change in expression upon translation limitation by Cm. (A and B) Gene operon composition as a function of protein sector. R indicates the R sector, NR indicates the other sectors. (C) Difference in the slope of protein concentration as a function of Cm between two genes of the same operon. The slope of the upstream gene is subtracted from the slope of the downstream gene. Note: slope values are not available for all genes in an operon. (D) Frequency of the difference between the arctangent function of the slope of the downstream gene and the upstream gene. (E) Frequency of change in slope sign between genes in the same operon. The sector partition and slopes used in panels A, C, and D are from Hui et al. (26). Download FIG S7, EPS file, 0.4 MB. Copyright © 2020 Zhang et al. 2020 Zhang et al. This content is distributed under the terms of the Creative Commons Attribution 4.0 International license.

10.1128/mSystems.00575-20.9 FIG S8 Estimation of the change in transcription and translation rate of the *gfpmut2* gene for each promoter construct. The first row is the same data as in Fig. 1 in the main text. The second row is the (relative) transcription rate (promoter activity), and the third row is the (relative) translation rate. Here, we assume that the translation rate for the GFP is the same independently of the promoter. The change in the amount of free RNAP was estimated from the ratio of PLtet to P5 as in Fig. 2 in the main text. This was used to estimate the transcription rate of P5-GFP expression from the dissociation constant *K*_5_. The transcription rate was then used to estimate the translation rate that results in the GFP expression rate data in the first row. This translation rate was then used to estimate the transcription rates for PLtet and P1. A similar result is obtained by estimating the transcription rates for PLtet and P1 from their RNAP-promoter dissociation constants. Download FIG S8, EPS file, 0.3 MB. Copyright © 2020 Zhang et al. 2020 Zhang et al. This content is distributed under the terms of the Creative Commons Attribution 4.0 International license.

## DISCUSSION

### A linear decrease in transcription capacity with increased translation limitation.

Here, we have used the approach developed by Hans Bremer and colleagues (22–25), using the comparison of the activity of promoters with different RNAP affinities to estimate the changes in the amount of free RNA polymerase under translation limiting conditions. When the growth rate is varied by using growth media with different nutrient content, the data are consistent with a decrease in the amount of free RNAP with decreasing growth rate (Fig. 2A), in agreement with previous estimates (34). However, the decrease in free RNAP when growth rate decreases due to increasing translation limitation has a steeper, linear slope (Fig. 2B; see also Fig. S2 in the supplemental material), suggesting that a decrease in transcription capacity could limit the maximum growth rate under these conditions.

Perhaps surprisingly, the translation rate increases in the presence of sublethal levels of antibiotics targeting ribosomes (Fig. S8) (19). This can result from a decrease in ppGpp relieving the inhibition it has on ribosome assembly and activity, thus activating the subpopulation of ribosomes that is stored in an inactive state (7, 8, 19, 40–43). The higher increase in translation rate in the growth media without cAA is consistent with a larger fraction of inactive ribosomes in the cells growing at these lower growth rates that can be quickly reactivated by a decrease in ppGpp concentration, not only for rapid adaptation to changes in local nutrient content but also to respond to the presence of growth inhibitors (19, 40–43). In rich growth media, when ppGpp levels are low, the potential of the cell to increase its translation capacity to respond to the presence of the inhibitor is limited by the smaller fraction of inactive ribosomes. In this case, a decrease in transcription capacity when translation is compromised could play an important role for the cell’s continued growth by mantaining balanced amounts of mRNA and proteins (19, 44).

The evidence provided here points to a possible cellular adaptation mechanism leading to a reduction in transcription capacity when ribosome activity is compromised. Decoupling of transcription and translation can have several deleterious effects, including mRNA degradation and R-loop accumulation that can interfere with DNA replication, causing genome instability and increased mutation rates (45). Moreover, this adaptation decreases the cost of transcription of untranslated mRNAs (46) and allows for more resources to be available for the synthesis of increased amounts of ribosomes to respond to the presence of translation inhibitors.

### A decrease in transcription capacity results in repartition of RNAP depending on promoter affinity.

The increase in translation rate in the presence of sublethal concentrations of chloramphenicol can help explain the similarity in the change in GFP concentration when it is expressed from the two promoters, P1 and P5, that are differentially regulated by ppGpp but with an equivalently high affinity for RNAP. This similarity also indicates that the decrease in ppGpp, while it does have an effect on the transcription from P1 (Fig. 2C), does not have a very large effect on the repartition of RNA polymerase between ribosomal and nonribosomal promoters, as the same decrease in the amount of free RNAP is observed in growth media where the cells contain different amounts of ppGpp (Fig. 2B and Fig. S2).

The repartition of RNA polymerase instead results from the competition among promoters for a smaller pool of available enzyme and therefore depends on the affinity of their interaction, as can be observed on the data obtained comparing GFP expression from the high-affinity P5 promoter with the lower-affinity PLtet promoter. The decrease in the amount of free RNAP estimated by measuring the ratio of GFP production rates from these two constitutive promoters (PLtet/P5) is about 10-fold, independently of the presence of amino acids in the growth medium (Fig. 2 and Fig. S2) and therefore of the change in ppGpp concentration.

The decrease in free RNAP could be due to different factors affecting the nonspecific interactions of the enzyme with the genome (23); however, the results obtained here from the comparison of the expression of two proteins of different lengths, β-galactosidase (β-gal) and GFP (Fig. 3) and of proteins encoded by genes within the same operon (Fig. 4 and Fig. S7) suggest that a decrease in the amount of the full-length protein may have a significant contribution to this effect. The RNA polymerase core contains two of the longest proteins in E. coli, the β and β' subunits (Fig. S6) that are found one after the other within the same operon, increasing the probability that a ribosome will stall before reaching the end of the mRNA. This is consistent with the results from a recent study by Zhu and coworkers showing that sublethal levels of chloramphenicol result in premature transcription termination and that this effect can also result in decreased expression of genes found downstream within a given operon (21).

This interpretation is further supported by the proteomics analysis of Hui et al. (26). They measured the change in protein fraction of more than 1,000 proteins in the presence of increasing concentrations of Cm by quantitative mass spectrometry. Their results show that the β and β' subunits of RNAP remain a constant fraction of the proteome with increasing Cm and decreasing growth rate. If the concentration of RNAP decreases with increasing Cm, and transcription is limiting for total protein production, then the amount of RNAP will determine the total amount of proteins that can be produced. The fraction of RNAP over all other proteins will thus remain nearly constant as chloramphenicol concentration is increased and growth rate decreases.

Moreover, these results can shed light on a recent study by Dai et al. (19) where it was proposed that in the presence of sublethal concentrations of Cm, despite an increase in the translation elongation rate due to a higher concentration of ternary complexes, the reduction in the total protein production rate results from a decrease in the active ribosome fraction, or the fraction of ribosomes that can reach the end of a mRNA in the presence of the inhibitor (19). Here, we identify RNAP as one of the genes that is likely to be most affected by the decrease in ribosome processivity due to the length of its β and β′ subunits, while shorter genes are affected to a lesser extent.

### Is the extreme length of RNA polymerase genes a feature conserved for the coupling of translation and transcription rates?

The extreme length of the β and β′ subunits of RNAP is conserved throughout bacteria (47). In the case of *Helicobacteraceae* and *Wolbachia*, the two genes are even fused together (47, 48). The gene length of β and β′ subunits can vary in different strains since they are composed of independent structural modules separated by spacers of differing length (47). In E. coli, the spacer sequences, which account for more than 25% of the total sequence, can be deleted without causing a significant decrease in transcription activity. In archea and chloroplasts, some of the conserved protein modules are found in separate genes, and in E. coli, they can be split from each other to produce an active enzyme (49), suggesting that the length of these genes is not imposed by functional constraints. The reason why the RNAP and ribosomal proteins find themselves at opposite ends of the spectrum of gene lengths in bacteria is likely linked to the assembly process, structural flexibility, and stability of the final multiprotein complex (50, 51); however, these results suggest that it could also play an important role for the cell’s continued growth with balanced amounts of mRNA and proteins in the presence of antibiotics that decrease translation processivity.

**FIG 5:**
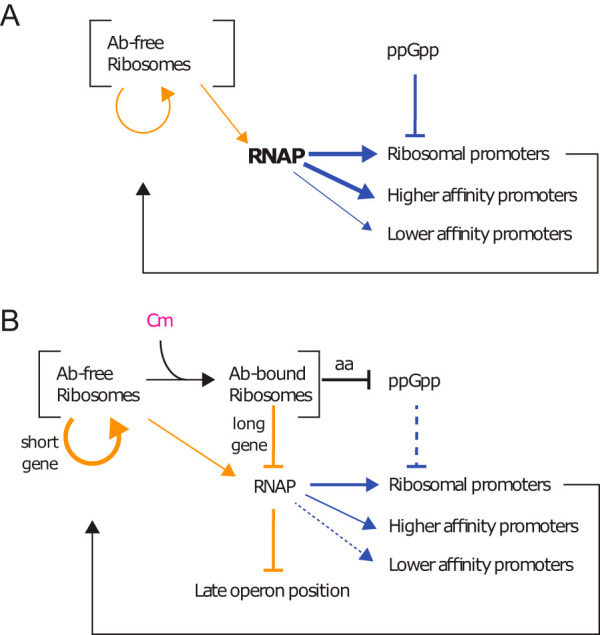
Summary. Orange arrows show translation effects, and blue arrows show transcription effects. (A) In the absence of translation limitation, the amount of RNAP available is regulated in part by its partition between ribosomal and nonribosomal promoters by the changes in ppGpp and the ensuing transcription rate of ribosomal promoters. (B) The decrease in ribosome processivity by inhibitors such as chloramphenicol (Cm) increases the probability of mRNA degradation, thus penalizing the expression of longer genes and of genes at the end of operons. RNAP subunits β and β' are among the longest genes in E. coli and are found one after the other within the same operon, while ribosomal proteins are among the shortest genes. The decrease in free RNAP can be measured by a decreasing ratio of high-affinity to low-affinity promoter gene expression rate. In nutrient-poor media, inhibition of ribosome activity by chloramphenicol increases the pool of amino acids and decreases the levels of ppGpp, increasing both ribosome production and ribosome activity. Ab, antibiotic.

The decrease in growth rate due to limiting transcription may be unexpected, as ribosome activity is usually thought to always be rate-limiting for bacterial growth; however, depending on the growth conditions, transcription has also been seen to become limiting in eukaryotic cells (68), pointing to different strategies of cellular adaptation to changing growth conditions and limitations. Understanding how bacteria modulate their growth rate and resource allocation in response to inhibition of growth has paramount importance in biotechnological and health applications (52–55). The results presented here provide a new cellular mechanism by which bacterial cells can decrease their growth rate in response to antibiotic stress (1, 44, 56). In summary, in the presence of sublethal concentrations of chloramphenicol, it is not translation that becomes limiting for the cell's growth rate, or the ppGpp-dependent repartition of RNAP between ribosomal and nonribosomal promoters, but it is the decrease in total transcription capacity (Fig. 5). It remains to be established whether this is a common response to other translation limiting factors, although a similar pattern of a decrease in growth rate despite a proportional increase in rRNA content and translation rate has been observed in the past with antibiotics such as tetracycline, erythromycin, and neomycin, and limiting expression of initiator factors 2 and 3 (18, 19).

## MATERIALS AND METHODS

### Strains, promoters, and reporters.

GFP and β-galactosidase (β-gal) were used as the reporter proteins to measure the rate of gene expression from a specific promoter. The GFP gene used is *gfpmut2* coding for a fast-folding GFP (57). The β-galactosidase gene is a 5′-end-modified *lacZ* from the pCMVbeta plasmid (58). Comparison with the wild-type *lacZ* gene shows that the additional 23 amino acids do not change the results obtained with this version of the reporter gene (data not shown). The promoters include two constitutive promoters (P5, obtained from T5 phage, and PLtet, i.e., P_LtetO-1_ [31]) and a shortened version of an rRNA promoter (*rrnB*P1 without the upstream Fis and H-NS sites) (see Table S1 in the supplemental material). The constructs of P1-*gfpmut2*, P5-*gfpmut2*, PLtet-*gfpmut2*, and P5-*lacZ* with a divergent kanamycin resistance gene were inserted in the chromosome of the Escherichia coli BW25113 strain. P1-*gfpmut2* and P5-*gfpmut2* (for Fig. 1) were inserted at position 258235 between the convergent *crl* and *phoE* genes, PLtet-*gfpmut2* was at position 356850 between *cynR* and *codA*, and P5-*lacZ* and P5-*gfpmut2* (for Fig. 3) were at position 1395689 between *uspE* and *ynaJ*. Genome position did not have an effect on the change in reporter gene expression as a function of Cm. The double fluorescent protein system (RFP-GFP constructs) has been described previously (39). The ribosome binding sites (RBS), i.e., Shine-Dalgarno sequences, used in the above constructs are all similar to the consensus UAAGGAGGU (59). The RBS for GFP (*gfpmut2*) and β-gal (*lacZ*) is GAAGGAGAU; for RFP (*mCherry*), it is AGAGGAGAA.

### Bacterial growth and fluorescence measurements.

Bacterial growth was carried out in M9 minimal growth medium supplemented with 0.5% glycerol (gly), 0.5% glucose (glu), 0.5% glycerol plus 0.2% Casamino Acids (cAA+gly) and 0.5% glucose + 0.2% Casamino Acids (cAA+glu). The preculture was obtained from the inoculation of one bacterial colony in LB growth medium. After overnight growth, the seed culture was washed once with phosphate-buffered saline (PBS) and diluted 200 times with the corresponding growth medium containing a specific concentration of chloramphenicol (Cm). This culture was diluted again 200 times once it reached exponential phase. The cultures were grown in flasks, shaking at 37°C and 170 rpm, and optical density and fluorescence were measured with a plate reader (Tecan Infinite 200Pro) every 30 to 50 min. Alternatively, the cultures were grown in a 96-well plate, with 150 μl of bacterial culture per well covered by 70 μl mineral oil. The culture plate was kept at 37°C in the plate reader, shaking and measuring fluorescence and optical density at 610 nm (OD_610_) every 7 min. The autofluorescence measured from the wild-type strain BW25113 was subtracted from the fluorescence of the fluorescent strains at the same OD (dependent on the medium). The experimental procedure of the β-galactosidase assay followed the protocol of Zhang et al. (60) except that the bacterial strains were cultivated in flasks instead of 48-well plates (18, 61, 62). The measurement of RFP-GFP constructs followed the protocol described previously (39).

### Analysis of GFP reporter expression data.

Experimental data obtained from the plate reader were analyzed with Matlab to obtain growth rate, protein concentration, and protein expression rate. The pipeline is shown in Fig. S1 in the supplemental material. The window in the growth curve corresponding to the exponential growth phase was defined as a linear range between an upper and a lower threshold in the growth curve plot of log OD_610_ versus time (the thresholds determined manually or from an automated method [63] gave similar results). Growth rate was derived from the slope of log OD_610_ versus time in exponential phase (Fig. S1B and C). GFP concentration was derived as the slope of the plot of GFP versus OD_610_ in the exponential growth phase (Fig. S1B). β-Galactosidase concentration in Miller units was obtained by the following formula (Fig. S1C):

(2) $$\text{β-gal activity}=1,000A\left(\frac{1}{0.01}\right)\left(20\right)\left(\frac{1}{{\text{OD}}_{610}}\right)=(2\times 10^{6})\left(\frac{A}{{\text{OD}}_{610}}\right)$$

where *A* comes from the fit of OD_450_ as a function of time with the formula *A*(1 – *e*^–γ^*^t^*)/γ and γ is a decay factor from taking into account that the reaction product *o*-nitrophenol is volatile (60). The rate of protein expression is defined as the product of protein concentration and growth rate (μ).

### Estimation of the change in RNAP concentration from the analysis of the effect of Cm on GFP expression from the P5 and PLtet promoters.

P5 and PLtet are constitutive promoters; therefore, their binding affinity with RNAP alone can be used to determine the probability that RNAP will be bound to the promoter at a given RNAP concentration. If the difference in RNAP affinity for two constitutive promoters is known, the difference in transcription rate from these promoters can be used to estimate the change in free RNAP concentration *in vivo* (Fig. 2 and Fig. S2). In order to obtain an estimate of the absolute concentration of RNAP, the data obtained on the transcription rates in the absence of Cm can be compared to previously published values as a function of growth rate (34).

Transcription initiation can be described by Michaelis-Menten kinetics, where the process of RNAP binding with the promoter is faster than the following isomerization steps including the formation of open complex. We formalize the relative transcription rates (TR) of P5 and PLtet as

(3) $$\text{TR}\left(\text{P}5\right)=c_{f}/\left(c_{f}+K_{5}\right)$$

and

(4) $$\text{TR}\left(\text{PLtet}\right)=ac_{f}/\left(c_{f}+K_{\text{Ltet}}\right)$$

where *c_f_* denotes the free RNAP concentration, *K*_5_ and *K*_Ltet_ are the dissociation constants for P5 and PLtet, respectively, and *a* is a scaling factor that accounts for the difference in transcription initiation frequency. The transcription initiation frequency, or promoter escape, is higher at PLtet than at P5, resulting in an increased probability of GFP expression for each binding event, and is assumed to be independent of RNAP concentration. This can explain why the GFP production rate (Gpr) PLtet-to-P5 ratio is greater than 1 (Fig. 2 in the main text and Fig. S3A), despite the difference in binding affinity.

The ratio of two dissociation constants (*K_j_*/*K_i_*) can be represented as an exponential function of the difference of the corresponding binding energies (*E_j_* – *E_i_*), i.e., $K_{j}/K_{i}=e^{E_{j}-E_{i}}$. The binding energies of RNAP with the three promoters can be estimated based on their DNA sequence (see above). If one dissociation constant (*K_i_*) is known, the other one, (*K_j_*), can be estimated with the formula $\;K_{j}=K_{i}e^{E_{j}-E_{i}}$.

We assume that the translation rate of GFP from the three promoter-*gfpmut2* constructs is the same, and that therefore it will cancel out when the ratio of GFP expression rate is taken. The ratio of GFP production rates for PLtet and P5 is thus equivalent to the ratio of the transcription rates (TR) obtained from equations 3 and 4

(5) $$\frac{\text{Gpr}\left(\text{PLtet}\right)}{\text{Gpr}\left(\text{P}5\right)}=\frac{\text{TR}\left(\text{PLtet}\right)}{\text{TR}\left(\text{P}5\right)}=a\frac{c_{f}+K_{5}}{c_{f}+K_{\text{Ltet}}}$$

If the difference in RNAP binding affinities for P5 and PLtet are known (from the free energy calculation above), the change in Gpr ratio with growth rate or with the Cm concentration can be used to estimate the change in free RNAP concentration (Fig. 2 in the main text).

It is also possible to estimate the absolute free RNAP concentration in the cell from the available data on the change in the concentration of free RNAP as a function of growth rate (34) and our data on the PLtet/P5 Gpr in different growth media. The free RNAP concentration, *c_f_*, as a function of growth rate (μ) can be obtained by fitting the data of Klumpp and Hwa (34), with log *c_f_* = *A*exp(–μ*_r_*/μ), giving *A* = 6.82log μm^−3^ and μ*_r_* = 0.11 doublings/hour (Fig. S2B). The change in Gpr(PLtet)/Gpr(P5) as a function of growth rate obtained by our experimental data (Fig. S2A) is consistent with the change in RNAP concentration measured previously (34). We can thus obtain the value of *a* and *K*_5_ by fitting the Gpr ratio data with equation 5 and the RNAP concentration at the different growth rates: *a* = 10.5 and *K*_5_ = 90 μm^−3^. Finally, we can use equation 5 to estimate the free RNAP concentration in the presence of Cm (Fig. S3B).
