# Supplementary material for: A Decrease in Transcription Capacity Limits Growth Rate upon Translation Inhibition
Source: mSystems. 2020 Sep 8;5(5):e00575-20. doi: 10.1128/mSystems.00575-20 (PMC7483510; doi:10.1128/mSystems.00575-20)
Supplement: TEXT S1 [file mSystems.00575-20-s0001.pdf]

Supplementary Information for “A decrease in transcription capacity limits growth rate upon translation inhibition”

Supporting Information Text

Estimation of the affinity of RNAP-promoter interaction from the promoter sequence. In 1987, Berg and von Hippel proposed a statistical-mechanical selection model to quantify the relationship between the affinity of a protein binding to a site on the DNA and individual base-pair choices in the site (1). They derived a formula to calculate the discrimination energy (E) for a specific sequence $\{B_l\}_{l=1}^{s}$:

$$E(\{B_l\}) = kT \sum_{l=1}^{s} \ln\left(\frac{n_{l0}+1}{n_{lB_l}+1}\right), \quad [1]$$

where k is the Boltzmann constant, T is the temperature, n_{lB_l} denotes the number of the sample sequences which have base B_l at position l, n_{l0} denotes the number of the sample sequences which have the cognate base at position l. To calculate the energy of RNAP binding to the promoter, we consider the contribution of the -10 and -35 sequences. The promoter sequences were obtained from RegulonDB (2). We consider the promoters recognized by sigma70 and the sequence of the 6 base pairs in each of the -10 and -35 regions. We obtained the frequency matrix from the literature (3), or by counting all the promoters under consideration. If we consider the bias of *E.coli* genome in base usage and the effect of the spacer length between -10 and -35 regions, equation 1 can be updated as (1)

$$E(\{B_l\}) = kT\left\{\sum_{l=1}^{s} \ln\left[\frac{(n_{l0}+1)p_{B_l}}{(n_{lB_l}+1)p_{l0}}\right] + \ln\left[\frac{n(L_{opt})+1}{n(L)+1}\right]\right\}, \quad [2]$$

where p_{B_l} is the occurrence probability of B_l in the genome, p_{l0} is the occurrence probability of the cognate base at position l in the genome, $n(L)$ is the number of the promoters with the spacer length L and L_{opt} is the optimal spacer length. The GC content of the *E.coli* genome is 43.4%. So the occurrence probabilities of A, T, C, G in the genome are 28.3%, 28.3%, 21.7% and 21.7% respectively. The dissociation constant (K) for one promoter (P1, P5, or PLtet) can be related to the corresponding binding energy (E) by $K \propto \exp(E)$.

Estimation of the change in ppGpp concentration from the analysis of the effect of Cm on GFP expression from the P5 and P1 promoters. P1, as a ribosomal RNA promoter, is inhibited by ppGpp. Therefore, its transcription rate is determined by both its binding affinity with RNAP and ppGpp concentration. ppGpp together with DksA mainly affect the isomerisation to the open complex rather than the promoter binding step (4, 5). The relative transcription rate (TR) of P1 can then be approximately formalized as

$$TR(P1) = F(c_p) \cdot c_f/(c_f + K_1), \quad [3]$$

where K_1 is the Michaelis constant and $F(c_p)$ is a function of ppGpp concentration (c_p). For simplicity, we assume

$$F(c_p) = b \cdot K_p^n/(c_p^n + K_p^n) \quad [4]$$

where K_p and n are microscopic dissociation constant and Hill coefficient for ppGpp inhibition, and b is a scaling factor.

According to the Materials and Methods in the Main text, the relative transcription rate (TR) of P5 can be expressed as

$$TR(P5) = c_f/(c_f + K_5) \quad [5]$$

The ratio of GFP production rates for P1 and P5 is equivalent to the ratio of the transcription rates obtained from Eqs. 5 and 3, and then the ratio of the GFP production rates for P1 and P5 can be expressed as

$$\begin{aligned} Gpr(P1)/Gpr(P5) &= TR(P1)/TR(P5) \\ &= b\frac{K_p^n}{c_p^n + K_p^n}\frac{c_f + K_5}{c_f + K_1}. \end{aligned} \quad [6]$$

So the effect of ppGpp inhibition can be represented by

$$bK_p^n/(c_p^n + K_p^n) = Gpr(P1)/Gpr(P5)/[(c_f + K_5)/(c_f + K_1)]. \quad [7]$$

In the absence of Cm, ppGpp concentration can be expressed as a function of growth rate, i.e. $c_p = c_{p0}\exp(-\mu/\mu_p))$, where c_{p0} and μ_p can be fitted with the data of Bremer and Dennis (6): $c_{p0} = 96.6$ pmol/OD_{460} and $\mu_p = 1.06$ doublings/hour. With this function, ppGpp concentration can be estimated at the different experimental growth rates. Since c_p and the right side of Eq.7 are known, we determine b, K_p and n by fitting with Eq.7: $b = 4.1$, $K_p = 42pmol/OD460$ and $n = 3.5$. We can then estimate the ppGpp concentration in presence of Cm (Fig. S2 C-D).

Estimation of the change in transcription and translation rates as a function of Cm. Using the determined K_5 and free RNAP concentration into Eq. 5, we can estimate the relative transcription rates (promoter activites) for P5. We use the ratio between GFP production rate and relative transcription rate (i.e. Gpr/TR) to indicate the relative translation rate. The relative translation rate is the same for all the three promoters according to the assumption, i.e. $Gpr(P5)/TR(P5) = Gpr(PLtet)/TR(PLtet) = Gpr(P1)/TR(P1)$. Then the common relative translation rate can be determined by $translation\ rate = Gpr(P5)/TR(P5)$. Finally, we estimate the transcription rates for PLtet and P1 by $TR(PLtet) = Gpr(PLtet)/translation\ rate$ and $TR(P1) = Gpr(P1)/translation\ rate$.

Fig. S8 shows that the partitioned Cm effects on GFP expression from three promoters at the transcription and translation levels. The differences between the results obtained with the three promoters constructs can be explained by the differences in RNAP affinity and the regulation by ppGpp of P1 transcription.

A quantitative model of the probability of a stalled ribosome as a function of gene length as a function of chloramphenicol concentration. Recently, Hwa and coworkers (7) have proposed a qualitative model to describe the effect of ribosome inhibition on protein synthesis which proposes that the stalling of the ribosomes on the mRNA results in an increased rate of mRNA degradation. A quantitative framework was developed to describe this process. We have started from this framework to develop a model to predict the effect of translation inhibition on the number proteins synthesized from a mRNA as a function of gene length and gene position in an operon.

The assumptions we adopt here are the same as Dai *et al.* (7): the first translating ribosome is coupled to RNA polymerase (8), and the consecutive flow of translating ribosomes along mRNA protects mRNA from degradation by RNase enzymes (9). The presence of a stalled ribosome results in an unprotected gap of the mRNA which is exposed to cleavage by RNaseE endonuclease (9–11). Inhibition of ribosome activity by Cm increases the stalling probability. The ribosomes behind the stalled one are released from the mRNA by the tmRNA/ArfA/YaeJ rescue pathways (12), and the newly exposed mRNA is further degraded by a 3'-exonuclease (11). The leading ribosomes that are not stalled complete translation, and the newly exposed mRNA is degraded (11, 13, 14).

Starting from the above sequence of events, we developed a quantitative model to try to explain the observed effects of Cm on protein expression as a function of gene length. Assuming that stalling is a Poisson process with rate β, the probability that one translating ribosome is stalled before time t is $f_{stall}(t) = 1 - \exp(-\beta t)$ under the initial condition $f_{stall}(0) = 0$. Notice that if the stall of a ribosome

is only caused by Cm hit, then the stall probability of a ribosome (f_{stall}) is equal to the probability of a ribosome hit by Cm (P_{hit}) proposed by Dai et al. (7). The probability that a ribosome is not stalled on a mRNA with length L (i.e. successfully completing translation) is then

$$1 - f_{stall}(L/v) = \exp(-\beta L/v), \qquad [8]$$

where v is translation elongation rate. The mRNA here should have a minimal nonzero length ($L_{\min}$), i.e. $L \geq L_{\min}$ since the ORF length close to zero is meaningless. Moreover, the stalling events can be also induced by the intrinsic nature of the mRNA sequence (e.g. the anti-Shine–Dalgarno sequence and pseudoknots) (reviewed in (12)), so the rate β has a minimal value in absence of Cm, while the presence of Cm increases it. Thus we represent β as a linear function of chloramphenicol concentration ([Cm]) by

$$\beta = \beta_0 + k[Cm] \qquad [9]$$

where β_0 indicates the stalling rate in the absence of Cm. We estimate $\beta_0 = 0.007s^{-1}$ by fitting the data of Tsung et al. (15) (Fig. S3 A). $L \geq L_{\min}$ and $\beta_0 > 0$ guarantee that $\exp(-\beta L/v) < 1$.

We consider that each translating ribosome has the same stalling probability. Moreover, We assume the stalling of a ribosome causes mRNA degradation with a probability of 100% by the mechanisms described above since the stalling time of a ribosome hit by Cm is usually long because of the strong binding affinity between Cm and ribosome (16). Thus the probability that n proteins are synthesized, in total, from one mRNA with length L (i.e. that the $n+1$th translating ribosome is stalled and induces mRNA degradation and translation abortion of the ribosomes following it) is:

$$p(n) = (1 - f_{stall}(L/v))^n f_{stall}(L/v) = \exp(-n\beta L/v)(1 - \exp(-\beta L/v)) \qquad [10]$$

The average number of proteins produced from one mRNA is:

$$\langle n \rangle = \sum_{n=0}^{\infty} n \exp(-n\beta L/v)(1 - \exp(-\beta L/v)) = \frac{1}{\exp(\beta L/v) - 1} \qquad [11]$$

Notice that, in principle, $\langle n \rangle$ cannot go to infinity because $L \geq L_{\min}$ (see above).

The ratio of protein production from a shorter mRNA (length L_1) to the one from a longer mRNA (length L_2) is

$$\frac{\langle n_1 \rangle}{\langle n_2 \rangle} = \frac{\exp(\beta L_2/v) - 1}{\exp(\beta L_1/v) - 1} \qquad [12]$$

In order to fit this equation with the experimental data, we need to express β and v as functions of growth rate (λ) or Cm concentration ([Cm]). Under translation limitation, the RNA to protein ratio (r) is negatively correlated with growth rate (λ) by

$$r = r_{max} - \lambda/k_n \qquad [13]$$

where r_{max} is the maximal RNA to protein ratio and k_n is the "nutritional capacity" reflecting the quality of the growth medium (17). The ribosome elongation rate (v) and r have a Michaelis-Menten relation:

$$v = \frac{v_{max} r}{r + K_M} \qquad [14]$$

where $v_{max} = 22a.a./s$ and $K_M = 0.11$ (7, 18). Thus v can be expressed a function of λ.

Furthermore, we need to estimate the relationship between $[Cm]$ and growth rate. From Dai et al. (7), the fraction of active ribosomes can be expressed as

$$f_{active} = \frac{N_{Rb}^{active}}{N_{Rb}} \propto \frac{\lambda}{v \cdot r} \qquad [15]$$

and the ratio of ribosomes not bound with chloramphenicol can be represented by

$$\frac{[Rb]_f}{[Rb]_{tot}} = \frac{K_D}{K_D + [Cm]}, \qquad [16]$$

where K_D is the dissociation constant which may also reflect the effects of Cm uptake and other processes. We assume

$$\frac{f_{active}}{f_{active}[0]} \approx \frac{[Rb]_f}{[Rb]_{tot}} \qquad [17]$$

where $f_{active}[0]$ indicates the active ribosome fraction in absence of Cm, since active ribosome fraction and free ribosome ratio decreases with Cm concentration similarly (7). Based on Eqs.13-17, we obtain a quantitative relation between growth rate and $[Cm]$

$$\lambda = k_n(r_{max} - 1/F([Cm])), \qquad [18]$$

where

$$F([Cm]) = -0.5(\frac{1}{K_M} - \frac{1}{r_{max}}) + \sqrt{\frac{1}{4}(\frac{1}{K_M} - \frac{1}{r_{max}})^2 + \frac{g(\lambda_0)}{k_n r_{max} K_M}\frac{K_D}{K_D + [Cm]}},$$

$$g(\lambda_0) = \lambda_0(r_{max} - \lambda_0/k_n + K_M)/(r_{max} - \lambda_0/k_n)^2,$$

and λ_0 is the growth rate in the absence of Cm. The dissociation constant K_D for Cm binding with ribosome measured in different labs varies from 0.5 to 5 μM (16). So we take K_D as a free parameter and fix it by fitting the empirical growth rate as a function of Cm concentration with Eq.18.

Greulich et al.(19) also derived a formula for growth rate as a function of Cm concentration based on a mathematical model which integrates the dynamics of the uptake and binding with ribosome of chloramphenicol and two empirical relationships of growth rate and ribosome fraction from Scott et al. (17), i.e.

$$[Cm] = \frac{2IC^*_{50}}{\widetilde{\lambda^*}}\left(-\widetilde{\lambda}^2 + \widetilde{\lambda} + \frac{\widetilde{\lambda^*}^2}{4\widetilde{\lambda}} - \frac{\widetilde{\lambda^*}^2}{4}\right) \qquad [19]$$

where $\widetilde{\lambda} = \lambda/\lambda_0$, $\widetilde{\lambda^*} = \lambda^*_0/\lambda_0$ and IC^*_{50}, λ^*_0 and λ_0 are parameters dependent on the growth medium.

Eq 12 gives the expression ratio of two genes of different lengths. Putting Eq. 9 into Eq.12, we obtain that the ratio increases with increasing Cm concentration. The equations 12-9 and 18 give a good fit to the experimentally determined expression ratio of GFP to β-galactosidase as a function of growth rate (see Fig. 3 in the main text and Fig. S3 B) within reasonable parameter values: $L_{lacZ}(L_2) = 1024\ a.a.$, $L_{gfp}(L_1) = 238\ a.a.$; $v_{max} = 22\ a.a./s$, $K_M = 0.11$ (7, 18); r_{max}=0.668, $\lambda_0 = 1h^{-1}$ (17); $\beta_0 = 0.007s^{-1}$ (fitted with the data of Tsung et al. (15)); $k = 0.0088(\mu M \cdot min)^{-1}$, $K_D = 1.5\mu M$, scale factor= 0.216 (fitted with the data of GFP vs lacZ). Replacing Eq. 18 with Eq. 19, the model can also give a good fit (Fig. S3 C-D) with two extra parameters (instead of K_D): $IC^*_{50} = 4.5\ \mu M$ and $\lambda^*_0 = 1.28\ h^{-1}$ (19).

A decrease in translation processivity can result in decreased expression of late operon genes. Transcription and translation are coupled via a physical interaction between RNA polymerase and the first ribosome translating the mRNA, which can be mediated by NusG or RfaH (20–23). Therefore, an additional factor that could decrease the expression of longer genes, and of late genes within an operon, in the presence of translation inhibitors is the loss of this RNAP-ribosome interaction exposing the mRNA for degradation and/or decreasing RNAP speed and processivity (8).

Previous work has shown that inhibiting ribosome activity with a higher concentration of antibiotics than was used here can result in decoupling of transcription and translation (8). To test whether these sublethal concentrations of Cm could have a similar effect on the RNAP-ribosome interaction, constructs were made where two genes of equal length coding for a red and a green fluorescent protein are placed one after the other within the same operon (Figure 4A). In this case there is an equal probability that a

ribosome will stall during translation of either gene. However, if the first ribosome translating the upstream gene is inhibited, the one in contact with RNAP, it will also decrease the probability that the downstream gene will be transcribed. Indeed, in these constructs we observe a decrease in the GFP to RFP ratio with increasing Cm. This could be either due to a decrease in transcription processivity from the loss of the RNAP-ribosome interaction or to an increase in the probability that the operon mRNA is degraded before RNAP finishes GFP transcription due to early termination of RFP translation, or a combination of both.

The formation of a terminator hairpin in the transcribed mRNA can be used to detect the presence of a ribosome-RNAP interaction (24). If a ribosome is bound on the mRNA as it is being extruded from RNAP, the terminator hairpin structure cannot fold. If inhibition of ribosome activity decouples translation from the ongoing transcription, then the RNA will be allowed to fold and transcription will stop. Transcription terminators of different strength have been inserted between the two genes (24). The efficiency of these terminators is determined by the distance between the stop codon of the upstream gene and the hairpin loop forming sequence. Termination will be less efficient when this distance is shorter. If transcription and translation are decoupled, the RNAP to ribosome distance will increase and we expect that the efficiency of the weaker terminators will increase with increasing Cm. However, we observe the same fractional decrease in GFP/RFP in all the constructs, independently of the distance from the stop codon, or of the presence of the terminator (Fig. S4). Therefore, it seems that at these low Cm concentrations, the probability of decoupling of transcription and translation is not significant enough to allow for the folding of the hairpin loop. The stalling of a ribosome however, independently of its interaction with RNAP, can result in an increased probability of mRNA degradation. In this case, the stalling of non-leading ribosomes, which are in greater number than the one interacting with RNAP and thus a more probable target, can decrease the lifetime of the operon's mRNA, decreasing the probability that translation will be completed at the *gfp* gene. These results can be reproduced by a model using the parameters values for the probability of translation termination obtained from the comparison in GFP vs LacZ translation (Fig. 3) and the terminator strengths measured in the previous study (24) (Fig. 4B).

The quantitative model of the probability of a stalled ribosome leading to mRNA degradation can also explain the effect of chloramphenicol on gene expression as a function of gene position in an operon. The above model of the probability of a stalled ribosome causing a decrease in further gene expression via mRNA degradation can also be used to explain the effects of Cm on gene expression related to the position of the gene in an operon. Because the upstream gene is always transcribed before the downstream gene, at first there will be more ribosomes on the upstream gene than on the downstream one. Moreover, a stalled ribosome on the upstream gene will stop transcription of the downstream gene. Therefore, there is a higher probability that a ribosome stalled on the upstream gene leading to the operon mRNA degradation will affect the expression of the downstream gene than *vice versa*. This only holds for the translation taking place during transcription. Once the operon mRNA is fully transcribed the ribosome density on the two genes will eventually become the same, with a delay that depends on the transcription rate and the translation initiation frequency. At this point there is an equal probability of hitting a RFP or a GFP translating ribosome. The stalling of a ribosome on the downstream gene will also result in the degradation of the operon mRNA, but it will decrease equally the translation of both genes and thus not affect the ratio between the two. The measurement of the ratio of the two proteins leads to the estimation of the probability that a ribosome in the upstream gene is hit before this equilibrium is reached. Here we consider the RFP-GFP system, shown in Fig. 4 in the main text, where the RFP gene is upstream of the GFP gene. The observed decrease in GFP/RFP with increasing Cm shows that ribosome stalling can take place before this equilibrium is reached and that its probability will increase as Cm is increased, as expected.

Let us set L equal to the length of the RFP gene and 2L the length of the whole operon. The probability that a ribosome is not stalled on a mRNA with length L (i.e. successfully completing translation) is $1 - f_{stall}(L/v) = \exp(-\beta L/v)$, where v is translation elongation rate and $\beta = \beta_0 + k[Cm]$ (see above). Notice that v increases with Cm concentration (7) (see Eqs. 13-14, and 18). At a given Cm concentration, β and v are fixed, and then the probability function $1 - f_{stall}(L/v)$ is fixed as well.

For simplicity, as done for the single gene case, we ignore the stochasticity in translation initiation and elongation, i.e. we assume the ribosomes bind to RBS and move along mRNA with a constant rate. Since the ribosome binding sites for RFP and GFP in our RFP-GFP operon system are both close to the consensus, we can assume that RFP and GFP have the same translation initiation rate (denoted by α) and the average number of translating ribosome on a mature mRNA is more than 1, i.e. $\alpha L/v >> 1$. We still adopt the mRNA degradation mechanism described by Dai et al. (7), just as was done above for a single gene: a translating ribosome hit by Cm will be stalled more than 10 min on average (16); a stalled ribosome will leave a gap in the mRNA, between it and the ribosome translating in front of it, to which RNase can bind to and induce mRNA degradation by the direct entry pathway (9, 10); the ribosomes following the stalled one will be stalled as well and finally all the stalled ribosomes will be released (12); the mRNA region upstream the stall site will be degraded instantaneously from the 3'- to the 5'-terminal by the 3' exoribonucleases (11); the leading unaffected ribosomes downstream of the stall site will finish the translation and the mRNA region behind them will be degraded in a 5'-monophosphorylated end dependent way (11, 13, 14).

We consider the ribosomes translating RFP and GFP as two "queues" and number each ribosome on the queue sequentially. Each stalled ribosome can lead to mRNA degradation. We denote the queue number of the stalled ribosomes as $n_1 + 1$ and $n_2 + 1$, for RFP and GFP respectively. The number of ribosomes that complete the translation of RFP and GFP, i.e. the number of mature RFP and GFP, are thus no more than n_1 and n_2, respectively. To determine the numbers of mature RFP and GFP the effect of the degradation from one gene coding region on the translation of another gene also needs to be considered.

We first consider the effect of mRNA degradation from a ribosome stalled on the RFP ORF on GFP translation. By the time n_1 ribosomes translating RFP have arrived at the end of the RFP ORF, n_1 ribosomes have also initiated the translation of GFP (on average). The degradation of the RFP coding region leads to the degradation of the GFP coding region because the free 5'-monophosphorylated end of the mRNA enhances RNaseE activity (13, 14). Thus, mRNA degradation from the RFP coding region limits the number of mature GFP to no greater than n_1. Therefore, the number of mature GFP equals $\min(n_1, n_2) \triangleq N_2$. Considering all the possible combinations of n_1 and n_2, we can derive the average number of mature GFP from one transcript by

$$\begin{aligned}\langle N_2 \rangle &= \sum_{n_1=0}^{\infty} \sum_{n_2=0}^{\infty} p(n_1) p(n_2) \min(n_1, n_2) \\ &= \frac{x^2}{1 - x^2}.\end{aligned} \qquad [20]$$

where $p(n) = \exp(-n\beta L/v)(1 - \exp(-\beta L/v))$ (see Eq. 10) and $x = 1 - f_{stall}(L/v) = \exp(-\beta L/v)$ (see Eq. 8). From Eq. 20, the average number of mature GFP from the operon is smaller than that in the case of GFP transcribed in the absence of the RFP coding region and Cm increases this difference. The degradation of mRNA from the upstream RFP region lowers the translation probability of the downstream GFP and the effect is higher with a higher Cm concentration.

Next we consider the effect of the degradation from the GFP ORF on RFP translation. When the $n_2 + 1st$ ribosome translating GFP is stalled, the number of ribosomes that have entered the GFP ORF is $n_2 + k$, where k is an integer from 1 to m ($\triangleq \alpha L/v$, where α is translation initiation rate, L is gene length, v is translation elongation rate). As mentioned above, m denotes the maximal number of translating ribosomes on the GFP ORF (with constant rates of translation initiation and elongation). The probability of k equal to each integer between 1 and m is the same. Since strong ribosome binding sites are used in our RFP-GFP operon system, we should have $m \gg 1$. The number of ribosomes translating RFP entirely should be the same as the number of ribosomes that have started to translate GFP, i.e. $n_2 + k$. Considering the degradation starting from both the GFP and RFP ORFs, the number of mature RFP should be $\min(n_1, n_2 + k)$ with a fixed k. Integrating all the possible values for k, the average number of fully synthesized RFP, is $\sum_k (\min(n_1, n_2 + k))/m \triangleq N_1$ (given n_1 and n_2). With all the combinations of

n_1 and n_2, finally, we can derive the average number of mature RFP from one transcript of the operon by

$$\begin{aligned} \langle N_1 \rangle &= \sum_{n_1=0}^{\infty} \sum_{n_2=0}^{\infty} p(n_1)p(n_2) \sum_{k=1}^{m} (\min(n1, n2+k))/m \\ &= \frac{x^2}{1-x^2} \left[1 + \frac{1}{x} - \frac{1}{m} \frac{1-x^m}{x(1-x)} \right] \\ &\approx \frac{x}{1-x}, \end{aligned} \qquad [21]$$

where $m \gg 1$ is used in the last derivation. From Eq. 21, the average number of RFP from the operon is approximately the same as in the case of RFP transcribed in the absence of the GFP coding region, suggesting the degradation from the downstream GFP has a small effect on the probability of translation of the upstream RFP.

Based on Eqs. 20 and 21, the ratio of the average numbers of mature GFP and RFP can be obtained as

$$\frac{\langle N_2 \rangle}{\langle N_1 \rangle} \approx \frac{x}{1+x} = \frac{1}{\exp(\beta L/v) + 1} \qquad [22]$$

If there is a terminator between the RFP and GFP genes, we assume its efficiency is y. RFP is expressed either from the middle-terminated transcript only including the RFP coding region or from the full transcript including both RFP and GFP coding regions, but GFP is only expressed from the latter transcript. So the ratio of the averaged GFP and RFP numbers is

$$\begin{aligned} \frac{\langle N_2 \rangle}{\langle N_1 \rangle} &\approx \frac{(1-y)\frac{x^2}{1-x^2}}{y\frac{x}{1-x} + (1-y)\frac{x}{1-x}} \\ &\approx (1-y)\frac{1}{\exp(\beta L/v) + 1}. \end{aligned} \qquad [23]$$

When β becomes larger due to translation limitation, the ratio $\langle N_2 \rangle / \langle N_1 \rangle$ decreases independently of the presence of a terminator. Fig. 4 in the main text shows that the ratios of the RFP and GFP levels obtained from experimental data can be fitted with the above model. The corresponding parameters are: $L_{gfp} = L_{rfp} = 238\ a.a.$; k_n=1.32, r_{max}=0.670, $\lambda_0 = 0.58h^{-1}$ (17); $v_{max} = 22\ a.a./s$, $K_M = 0.11$, $\beta_0 = 0.007s^{-1}$, $K_D = 1.5\mu M$,$k = 0.0088(\mu M \cdot min)^{-1}$ (the same values as those above obtained from the fit of the data of GFP vs lacZ); scaling factor=7.82 (fitted); terminator efficiencies of ter 1-4 are 0.167, 0.235, 0.526, and 0.837, respectively, the same as those measured previously (24).

Generality of the results. In order to determine whether the results obtained here, with this small set of promoters and reporter proteins, can be applied to the results obtained on larger datasets, we carried out an analysis of the effect of gene length and operon position on gene expression in the presence of increasing concentrations of Cm on the dataset from Hui and coworkers (25). In their study the amounts of more than one thousand proteins was measured as a function of increasing Cm concentration and under different growth limitations. The proteins were divided in three subsets on whether their concentration increased (R sector), remained the same or decreased (P sector) with increasing translation limitation (Fig. S5 A).

The first step is to determine how many of the genes in the R sector are influenced directly and indirectly by a change in ppGpp concentration. To do this we used the transcriptomics data on ppGpp dependent regulation from Traxler *et al.* (26). The comparison of these two datasets shows that about 50% of proteins in the R sector are expressed from genes negatively regulated by ppGpp (Fig. S5 B). In order to determine whether this was a direct or indirect regulation, we analyzed the promoter sequences. Promoters that are negatively regulated by ppGpp have a GC-rich sequence between positions -8 and -1, called the discriminator region (27). This analysis shows that the promoters from the subset of genes inhibited by ppGpp indeed tend to have a better match to the discriminator sequence than the others (Fig S5 C).

Therefore, ppGpp-dependent transcription regulation can explain the change in expression of a subset of R sector genes.

Promoter sequences can also be analyzed to estimate RNAP affinity using the statistical-mechanical selection method of Berg and von Hippel (1) (see details as above). This analysis shows that the R sector proteins tend to have promoter sequences with a higher affinity for RNAP, with a peak in the distribution below 5 kT (blue, red and purple lines in Fig. S6 A), than those of the other sets, having a wider distribution with a peak at about 5 kT (Fig. S6 A). The presence of a transcription factor can compensate for a low promoter affinity, however regulation by a transcription factor (as defined in RegulonDB (2)) does not change the repartition between R sector and non-R sector promoters. If ribosomal genes are taken out of the analysis the peak below 5 kT is lost (Fig. S6 B). The estimated relative affinities of the three promoters used here are shown on the same graph. All three are below 5 kT, among the higher affinity promoters.

We next looked at the protein length distribution in the different protein subsets. The genes in the R sector inhibited by ppGpp have a distribution with a peak below 600 nucleotides (Fig. S6 C, green line). These tend to be for the most part ribosomal proteins or proteins that regulate ribosome activity (Fig S6 E). The length of the *gfpmut2* and *lacZ* genes, as well as of the β and β' subunits of RNAP are shown for reference. The magnitude of the change in the protein concentration as a function of Cm in the Hui *et al.* dataset was measured by the slope of the data in the plot of the change in protein fraction vs growth rate (Fig. S5 A). The plot of the distribution of the slope values within different windows of gene length shows a more negative slope for the shorter genes (Fig. S6 D). Once the ribosomal genes are taken out of the analysis, the median slope increases, particularly for the genes around 300 bp long (Fig. S6 F). The distribution of the slope value as a function of gene length retains the same overall shape independently of whether the promoters are regulated by ppGpp, regulated by transcription factors or constitutive (data not shown). In summary, ribosomal proteins tend to be shorter than average, have higher affinity promoters and be negatively regulated by ppGpp, all factors that can lead to increased expression upon translation limitation in different growth media.

Finally, we carried out an analysis of the effect of a gene's position within an operon (Fig. S7). Despite sharing the same promoter region, genes in the same operon do not always belong to the same sector, as measured by the value of the slope in the protein fraction vs growth rate plot (Fig. S7 A, B). Within a given operon, the occurrence of early genes in the R sector and a downstream gene in the P sector is more frequent than the reverse case (binomial one-sided P-value: 3.2×10^{-5}) (Fig. S7 C-E). Consistent with the results obtained here with the RFP-GFP construct (Figure 4 in the main text).

The role of the discriminator region in determining changes of gene expression under translation limiting conditions. We asked how many of the proteins that have been identified as being part of the R-sector may be directly regulated by (p)ppGpp. In a previous study Hui *et al.* used mass spectrometry to measure the change in concentration of about 1000 different proteins in *E. coli* in the presence of different concentrations of Cm (25). They were thus able to identify which proteins change in concentration as R sector proteins and those that belong to the other sectors, because they are induced under different kinds of limitations. In another study Traxler *et al.* used microarrays to measure the changes in mRNA abundance when (p)ppGpp is either absent from the cells or is increased by the addition of serine hydroxamate (26). A Venn diagram shows that there is some overlap between the genes that were negatively regulated by (p)ppGpp and both R-sector and other sector proteins (Figure S5 B-C). We next asked how many of the genes in these subsets are regulated by promoters containing a discriminator sequence. We compared the sequence found between -8 and -1 of a gene's promoter with the one of the *rrnB*P1 promoter thus generating a "discriminator score". While all the subsets had a higher score than a set of random sequences, the subset that is at the intersection of the R-sector proteins and the genes showing an inhibitory effect of (p)ppGpp has a higher score. These results indicate that within the R-sector proteins there are both direct and indirect effects of (p)ppGpp on gene expression and that the direct effects are likely due to the presence of a discriminator region at the promoter.

Discriminator score. The discriminator score for each available promoter was calculated as follows. The discriminator region required for ppGpp inhibition is a GC-rich sequence between positions -8 and -1 of the promoter sequence relative to the transcription start site (28, 29). The study of Haugen et al. (30) had shown that the C at -7 is very important for the effect of (p)ppGpp, but that identity of the base at the -3 site is not significant. We therefore assumed a consensus sequence for the discriminator region as

-8	-7	-6	-5	-4	-3	-2	-1
c	C	c	c	c	-	c	c .
g		g	g	g		g	g

Based on this sequence, we scored each promoter by calculating the similarity of one promoter to this consensus sequence at positions -8 to -1. For *rrnB*P1 the score is 1. If a gene has more than one promoter, we take the highest score as its discriminator score.

Analysis of data obtained from the literature. We analyzed previously published data from the literature to test the generality of our results. The slopes of the change in protein levels under translation limitation and the sector partition of the proteome were obtained from Hui et al. (25). The lists of the genes inhibited and uninhibited by ppGpp were obtained from Traxlex *et al.* based on their microarray transcription data of the wild type and ppGpp0 (relA-spoT-) strains compared to cells under isoleucine starvation (26). The information on promoter sequences, gene lengths and gene positions in operons was obtained from RegulonDB (2).

References

1. Berg OG, von Hippel PH (1987) Selection of DNA binding sites by regulatory proteins. Statistical-mechanical theory and application to operators and promoters. *Journal of Molecular Biology* 193(4):723–750.
2. Gama-Castro S, et al. (2016) Regulondb version 9.0: high-level integration of gene regulation, coexpression, motif clustering and beyond. *Nucleic acids research* 44(D1):D133–D143.
3. Harley C, Reynolds R (1987) Analysis of E. coli promoter sequences. *Nucleic Acids Res* 15:2343–61.
4. Barker MM, Gaal T, Josaitis CA, Gourse RL (2001) Mechanism of regulation of transcription initiation by ppGpp. I. Effects of ppGpp on transcription initiation in vivo and in vitro11edited by R. Ebright. *Journal of Molecular Biology* 305(4):673–688.
5. Paul B, Berkmen M, Gourse R (2005) DksA potentiates direct activation of amino acid promoters by ppGpp. *Proc Natl Acad Sci U S A* 102:7823–8.
6. Bremer H, Dennis P (1996) Modulation of Chemical Composition and Other Parameters of the Cell by Growth Rate in *Escherichia coli and Salmonella typhimurium: cellular and Molecular Biology*, eds. Neidhardt F, et al. (American Society for Microbiology, Washington D.C.), 2nd edition, pp. 1553–1569.
7. Dai X, et al. (2016) Reduction of translating ribosomes enables Escherichia coli to maintain elongation rates during slow growth. *Nature Microbiology* 2:16231.
8. Proshkin S, Rahmouni AR, Mironov A, Nudler E (2010) Cooperation between translating ribosomes and RNA polymerase in transcription elongation. *Science* 328(5977):504–508.
9. Deana A, Belasco JG (2005) Lost in translation: the influence of ribosomes on bacterial mRNA decay. *Genes & development* 19(21):2526–2533.
10. Liang ST, Ehrenberg M, Dennis P, Bremer H (1999) Decay of rplN and lacZ mRNA in Escherichia coli. *Journal of Molecular Biology* 288(4):521–538.
11. Mackie GA (2013) RNase E: at the interface of bacterial RNA processing and decay. *Nature Reviews. Microbiology* 11(1):45–57.
12. Keiler KC (2015) Mechanisms of ribosome rescue in bacteria. *Nature Reviews Microbiology* 13(5):285–297.
13. Mackie GA (1998) Ribonuclease E is a 5'-end-dependent endonuclease. *Nature* 395(6703):720–724.

14. Callaghan AJ, et al. (2005) Structure of Escherichia coli RNase E catalytic domain and implications for RNA turnover. *Nature* 437(7062):1187–1191.
15. Tsung K, Inouye S, Inouye M (1989) Factors affecting the efficiency of protein synthesis in Escherichia coli. Production of a polypeptide of more than 6000 amino acid residues. *The Journal of Biological Chemistry* 264(8):4428–4433.
16. Harvey RJ, Koch AL (1980) How partially inhibitory concentrations of chloramphenicol affect the growth of Escherichia coli. *Antimicrobial Agents and Chemotherapy* 18(2):323–337.
17. Scott M, Gunderson CW, Mateescu EM, Zhang Z, Hwa T (2010) Interdependence of cell growth and gene expression: origins and consequences. *Science* 330:1099–102.
18. Klumpp S, Scott M, Pedersen S, Hwa T (2013) Molecular crowding limits translation and cell growth. *Proceedings of the National Academy of Sciences* 110(42):16754–16759.
19. Greulich P, Scott M, Evans MR, Allen RJ (2015) Growth-dependent bacterial susceptibility to ribosome-targeting antibiotics. *Molecular systems biology* 11(3):796.
20. Demo G, et al. (2017) Structure of RNA polymerase bound to ribosomal 30s subunit. *eLife* 6:e28560.
21. Fan H, et al. (2017) Transcription–translation coupling: direct interactions of rna polymerase with ribosomes and ribosomal subunits. *Nucleic acids research* 45(19):11043–11055.
22. Kohler R, Mooney RA, Mills DJ, Landick R, Cramer P (2017) Architecture of a transcribing-translating expressome. *Science (New York, N.Y.)* 356(6334):194–197.
23. Artsimovitch I (2018) Rebuilding the bridge between transcription and translation. *Molecular Microbiology* 108(5):467–472.
24. Li R, Zhang Q, Li J, Shi H (2016) Effects of cooperation between translating ribosome and RNA polymerase on termination efficiency of the Rho-independent terminator. *Nucleic Acids Research* 44(6):2554–2563.
25. Hui S, et al. (2015) Quantitative proteomic analysis reveals a simple strategy of global resource allocation in bacteria. *Molecular systems biology* 11(2):784.
26. Traxler MF, et al. (2008) The global, ppGpp-mediated stringent response to amino acid starvation in Escherichia coli. *Molecular microbiology* 68(5):1128–1148.
27. Haugen SP, Ross W, Gourse RL (2008) Advances in bacterial promoter recognition and its control by factors that do not bind DNA. *Nature Reviews. Microbiology* 6(7):507–519.
28. Travers AA (1980) Promoter sequence for stringent control of bacterial ribonucleic acid synthesis. *Journal of bacteriology* 141(2):973–976.
29. Travers AA (1984) Conserved features of coordinately regulated E. coli promoters. *Nucleic acids research* 12(6):2605–2618.
30. Haugen SP, et al. (2006) rRNA promoter regulation by nonoptimal binding of sigma region 1.2: an additional recognition element for RNA polymerase. *Cell* 125(6):1069–1082.
